# Radiation Damage of Myoglobin Crystals in Weak Stationary Electric and Magnetic Fields

**DOI:** 10.1088/1742-6596/493/1/012029

**Published:** 2014-03-13

**Authors:** C B Trame, M Dragovic, H-J Chiu

**Affiliations:** 1 Joint Center for Structural Genomics (http:/www.jcsg.org), USA; 2 Stanford Synchrotron Radiation Lightsource, SLAC National Accelerator Laboratory, Menlo Park, CA 94025, USA

## Abstract

Radiation damage is one of the bottlenecks in the field of structural biology. Cryo-cooling of protein crystals provided a breakthrough in the 1980s and resulted in significant reductions in radiation damage. Other factors positively influencing the progression of damage include the application of radical scavengers and reductions in the experimental beam size. Here we study the impact on radiation damage of applying static magnetic and electric fields during protein diffraction experiments, ultimately probing the Lorenz force effect on primary photoelectrons and secondary Auger electrons, which both contribute to the damage process. The design of a special mounting pin using graphene for applying electric fields on a crystalline sample is described. Analyses of myoglobin protein crystals exposed to the fields of ~40 mT and −300 V show a slower global radiation damage rate and also changes in the progression of specific damage process on the molecular level, in particular at doses extending beyond the Garman limit of 30 MGy.

## 1. Introduction

Myoglobin is one of the most studied biological molecules, especially in regard to radiation damage using X- and γ-rays [1]. The consequences of exposure to high energy photons on protein crystals, are specific structural changes, which lead to changes in global crystallographic parameters (I/σ(I), R_cryst_, mosaicity, anomalous signal and lattice dimensions). The structural changes are sensitive to chemical environment and are influenced by the distribution of photo- and Auger-electron cascades released in the photo-absorption process. It is the charge properties and the distribution of the resulting electrons and ions within protein crystals that prompted us to study the radiation damage effect in electric and magnetic fields. In addition, electric and magnetic fields are also known to influence protein crystallization process [2]. Qualitative crystallographic analyses of myoglobin crystals mounted on a newly designed pin using graphene support compared to crystals mounted on standard nylon loops suggests an improved crystal mount and data quality for protein diffraction experiments.

## 2. Experiment

**Figure 1:**
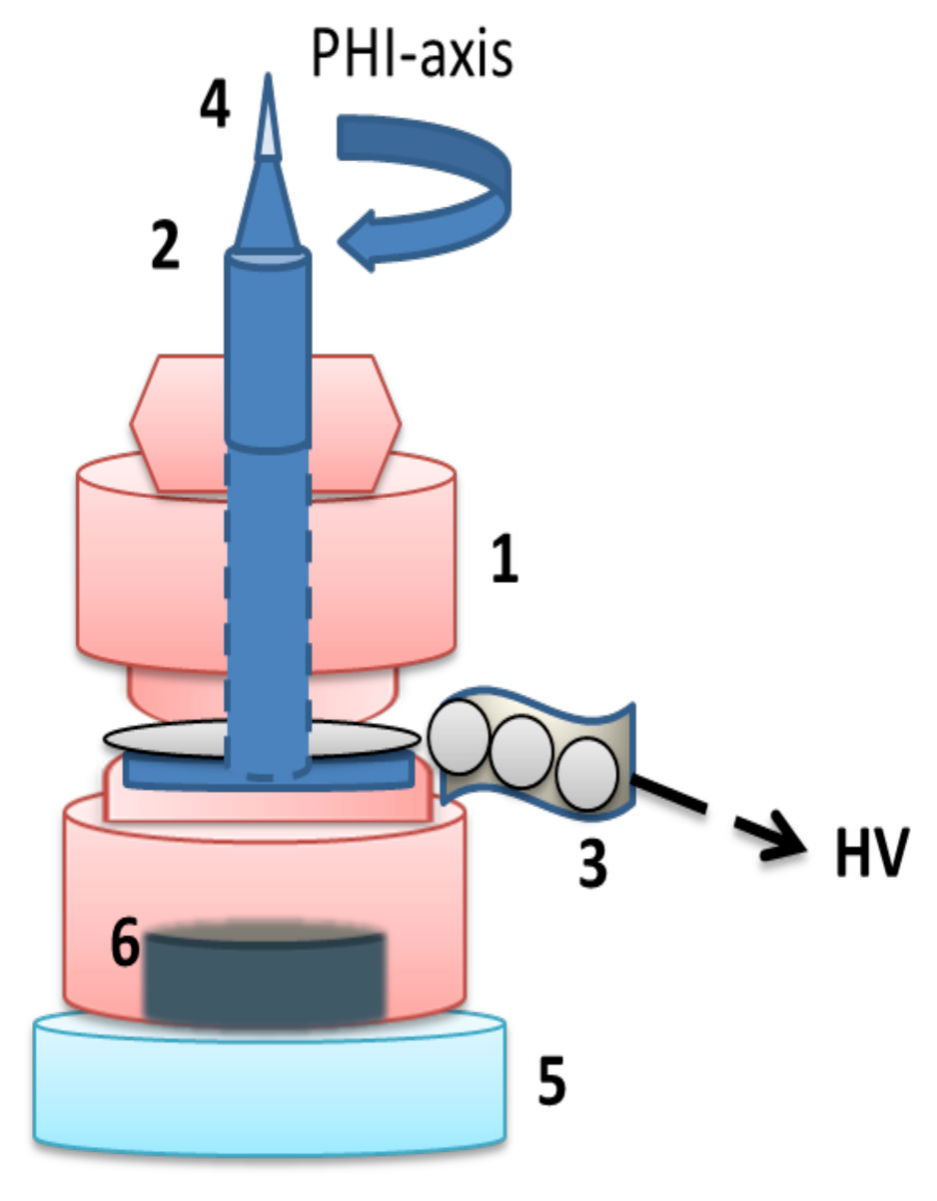
HV pin consisting of insulating plastic body (1) and a magnetic stainless steel core (2) glued to a thin disk, which is attracted to a flexible chain of magnetic beads inserted in a loose shrinking tube (3). The pin core and the tip with the graphene foil (4) are connected via low ohm conducting beads to the HV power supply (Model Ortec556). The pin is positioned on the goniometer base (5) via permanent magnet (6) and is centered in the middle of the 100K cold LN2 stream.

A special HV (High Voltage) pin was designed in order to apply electrostatic fields on protein crystals during diffraction experiments. Figure 1 shows the scheme of the design, which allows a free phi-rotation, while keeping a constant bias voltage on the crystal. For the application of magnetic fields, a rare earth permanent bar magnet (N52 type) was placed ~3 cm away from the sample, with its N or S pole oriented at ~35° to the phi axis. The stationary magnetic field was determined to be ~0.04 T around the crystal mounting graphene surface using 410 Lake Shore Gaussmeter, and the value varied by ~2% due to the background magnetic fields from other mounting components (3, 5 and 6).

**Figure 2:**
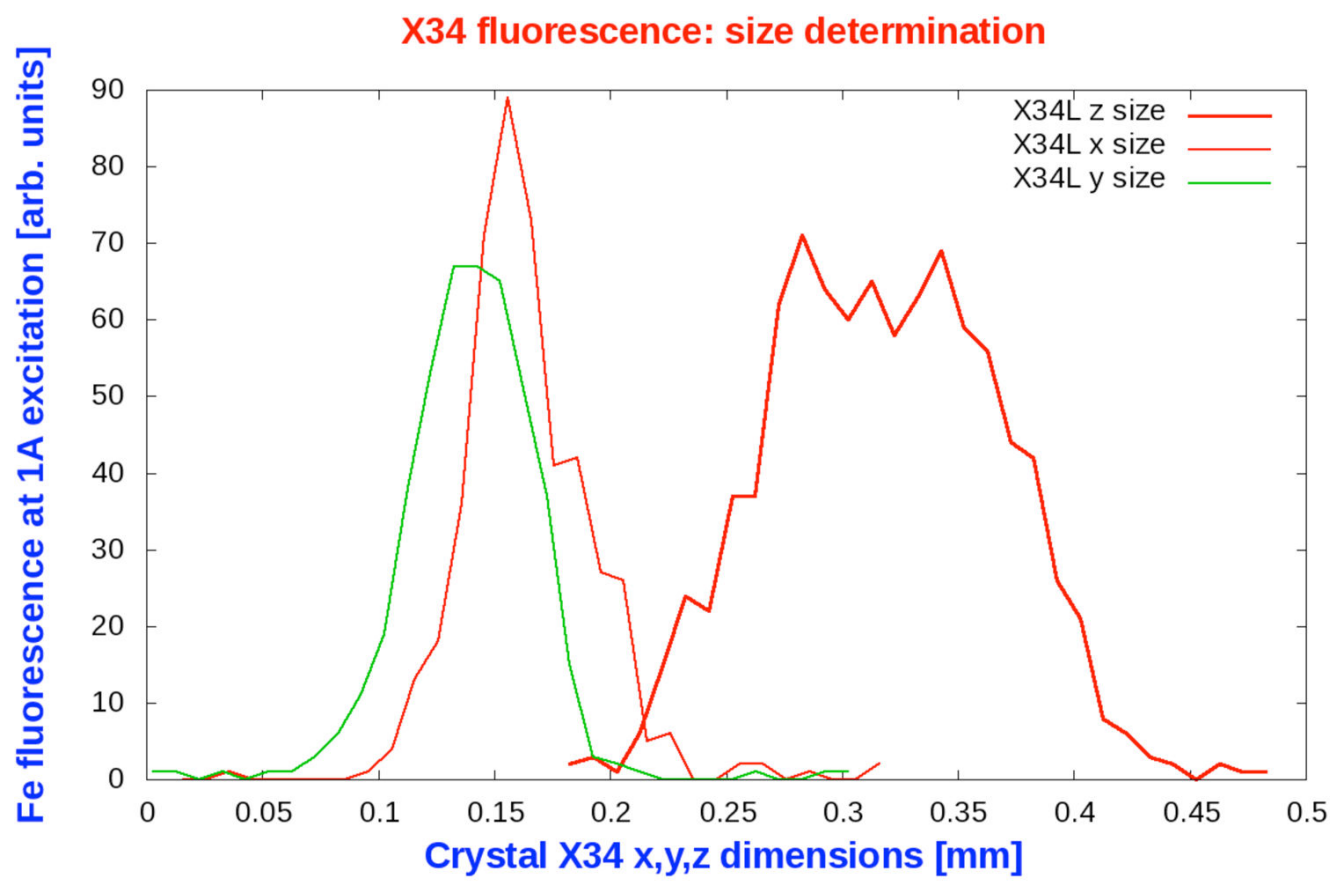
Measured dimensions of myoglobin crystal X34 in (x,y,z) as a profile of an integrated Fe fluorescence signal using a VORTEX Si-drift detector. The overall dimensions of ~ 175×101×93 μm^3^ match well with the size determined by a microscope camera. Signal to noise (loop+crystal/loop) ratio of this method is 50×60 with the beam sizes of (30×30)μm.

Sperm whale myoglobin was crystallized according to previously published methods [3]. All experiments were performed on the SSRL bending magnet beamline 14-1. Multiple data sets were collected from six myoglobin crystals (Table 1). All crystals diffracted to ~1.00–1.25 Å. Typically 10 data sets in the same 120° phi range for each crystal were collected at 1.0 Å wavelength and at 100 K. All data sets were processed using identical XDS data processing parameters. For the purpose of comparing radiation damage under different parameters, we chose to monitor 1.9–2.0 Å resolution shell. The crystal sizes were determined using a Navitar Lens based microscope camera. The size of each loop mounted crystal was further verified using Fe fluorescence signal integrated around Fe Kedge with the excitation wavelength of 1.0 Å. An example of fluorescence profile used to determine crystal size is shown in Figure 2.

With a photon flux of 1.02×10^11^ photons/sec at 1.0 Å wavelength, the program RADDOSE [4] calculated a radiation dose of ~4.5 MGy per data set. Typically 10 data sets were collected until a ~10-fold fall-off in I/σ(I) in the 1.9–2.0 Å resolution shell was reached. The experimental dose limit of 30 MGy was reached during the ~6^th^ – 7^th^ data set for each crystal. Due to the variation in photon flux and crystal size in different experiments, the scaling factor S, proportional to X-ray dose, was calculated using the general formula [5] and was applied to each data set:

$$S\propto (I_{o}/\lambda V)[1-\exp(-\mu_{\text{abs}}t)]$$

**Figure 3:**
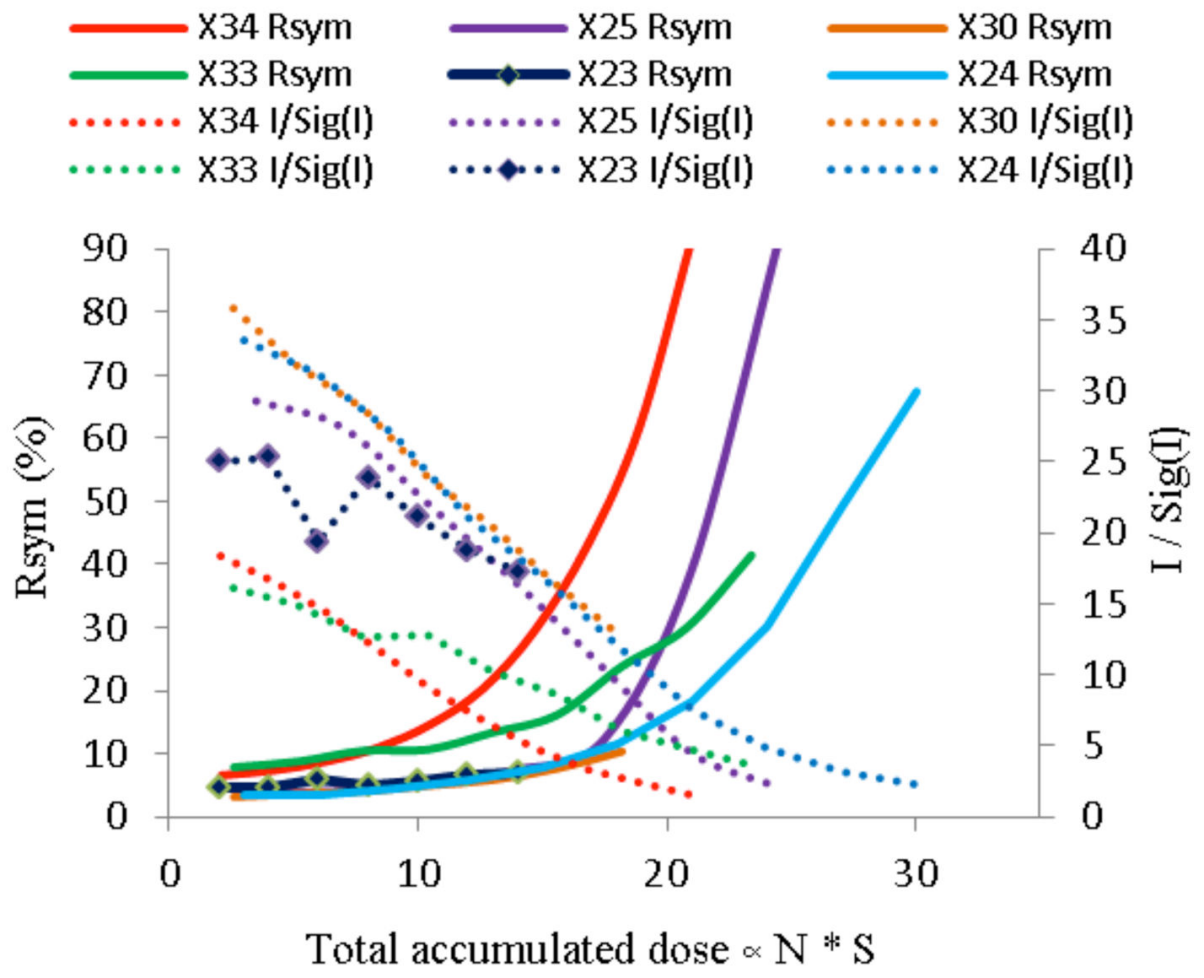
The increase of Rsym and the decrease of I/σ(I) as a function of accumulated X-ray dose show that the data quality deteriorates faster in crystals X25, X30 and X34, which were mounted in standard nylon loops, as compared to crystals X23 and X33, which were mounted on graphene foil and collected in the presence of magnetic and electric fields.

Here S is proportional to the absorbed energy per unit mass by a crystal with thickness t and volume V. The absorption coefficient μ_abs_ is calculated for an incident wavelength λ and beam intensity of I_o_. That crystal dependent dose scaling factors S, ranging between 2 and 3.5, were then multiplied by the data set number N for each of the crystals, and used as an abscissa in figure 3.

## 3. Results and Discussion

The XDS data reduction resulted in the fitted crystallographic parameters indicating the radiation damage progression for each of the crystals, as shown in figure 3.

**Figure 4:**
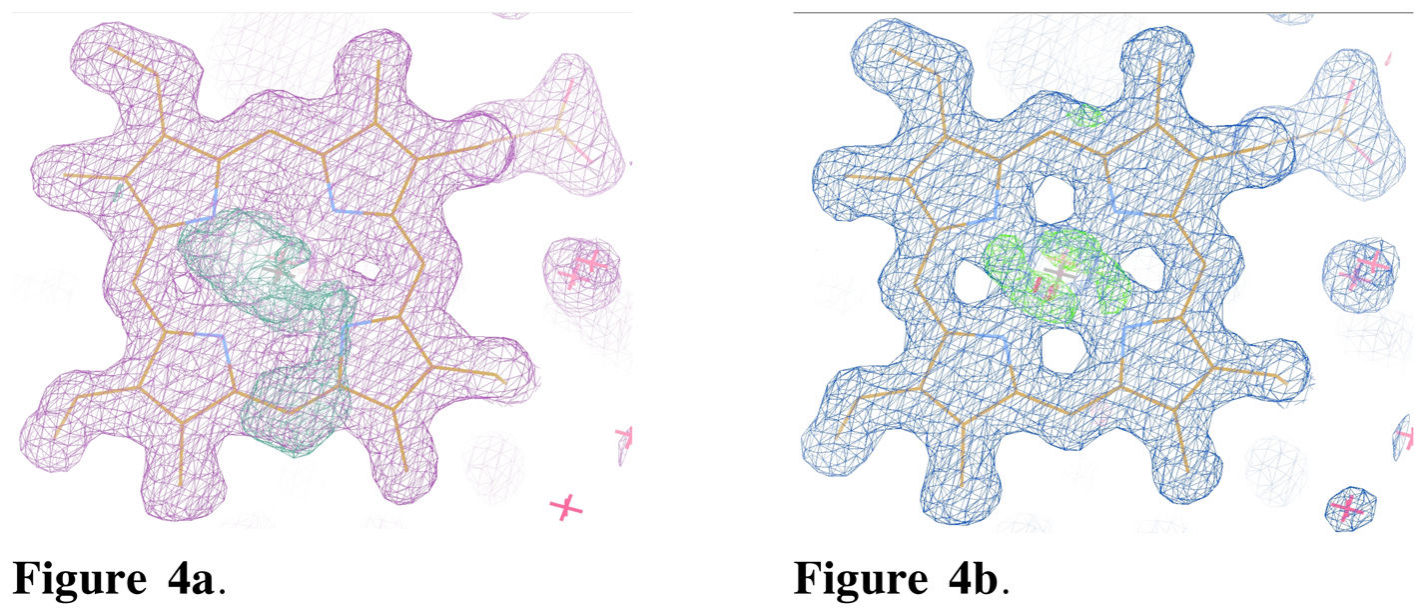
**Figure 4a.** Heme group in the 8^th^ data set of X34. The 2Fo-Fc map is colored in magenta and contoured at 1.1σ. The Fo-Fc maps are shown in green and red and are contoured at ±3.5σ. There is more positive Fo-Fc density spread out around the heme plane in X34 than in X33. **Figure 4b.** Heme group in the 8^th^ data set of X33. The 2Fo-Fc map is colored in blue and contoured at 1.1σ. The Fo-Fc maps are shown in green and red and are contoured at ±3.5σ. The positive Fo-Fc density is centrally localized around Fe atom. The Fe atom stays in the heme plane.

Our preliminary results suggest that myoglobin crystals suffer radiation damage at a slower rate when both electric and magnetic fields are applied simultaneously. Mounting crystals on a graphene surface also has the advantage of reducing radiation damage as compared to Hampton-style nylon loops. Specific structural damage at the atomic level reveals itself through density differences around the heme prosthetic group and charged residues, when comparing for example electron density maps of the 8^th^ set of X33 and X34 (Figure 4a and 4b).

The reduced amount of solvent/water due to surface tension effects for graphene foils over nylon loops may be a contributing factor in reducing radiation damage progression. The fact that the observed dose dependent effects are small at the beginning and become more apparent towards the end of data collection is possibly due to limited range of electromagnetic fields that are available in our current experimental setup. For future experiments, the hardware setup will be improved to allow higher electric and magnetic fields to be applied to the samples. It appears, that freezing myoglobin crystals in the presence of a magnetic field has a negative effect on the crystal, which tends to split. Similar tendency in worsening the diffraction pattern was observed with the application of +300V field in combination with B fields (hence data not shown). A fluorescence size profile scan was proved to be a useful tool for measuring the crystal size.

## Figures and Tables

**Table 1:** List of myoglobin crystals used in this study

Crystal ID.	Pin type	Mount	Operation sequence^a^
X23	HV	Graphene	HV −300V / B(N) / B(S) c
X24	HV	Graphene	B(S)
X25	Cu^b^	Nylon loop	--
X30	Cu^b^	Nylon loop	--
X33	HV	Graphene	HV −300V / B(S)
X34	Cu^b^	Nylon loop	--

a In the cases that both electric field and magnetic fields were applied during the data collection, the electric field was applied first and followed by magnetic field. B(N) and B(S) mean that the north or south poles face sample respectively.

b Copper pin from Hampton Research.

c The magnetic polarity B(N) was changed to B(S) after 3^rd^ data set.
